# Polydatin Inhibits Cell Viability, Migration, and Invasion Through Suppressing the c-Myc Expression in Human Cervical Cancer

**DOI:** 10.3389/fcell.2021.587218

**Published:** 2021-04-12

**Authors:** Longchang Bai, Yingkang Ma, Xue Wang, Qiongni Feng, Zhining Zhang, Sijie Wang, Huijie Zhang, Xinyu Lu, Yonghui Xu, Erhu Zhao, Hongjuan Cui

**Affiliations:** ^1^State Key Laboratory of Silkworm Genome Biology, College of Sericulture, Textile and Biomass Sciences, Southwest University, Chongqing, China; ^2^Cancer Center, Medical Research Institute, Southwest University, Chongqing, China; ^3^Westa College, Southwest University, Chongqing, China; ^4^Chongqing General Hospital, University of Chinese Academy of Sciences, Chongqing, China; ^5^Chongqing Engineering and Technology Research Center for Silk Biomaterials and Regenerative Medicine, Chongqing, China; ^6^Engineering Research Center for Cancer Biomedical and Translational Medicine, Southwest University, Chongqing, China

**Keywords:** polydatin, cervical cancer, c-Myc, cell viability, migration and invasion

## Abstract

Polydatin, an active ingredient from the roots of *Polygonum cuspidatum*, is considered to have protective effects on the cardiovascular system and liver. In this study, we demonstrated that polydatin has antitumor activity against human cervical cancer. Polydatin efficiently inhibited cervical cancer cell proliferation by regulating cell cycle-related proteins including p21, p27, CDK2, CDK4, Cyclin D1, and Cyclin E1. Furthermore, polydatin suppressed cell invasion and migration by regulating epithelial–mesenchymal transition (EMT) markers, including E-cadherin, N-cadherin, Snail and Slug. The c-Myc, as a proto-oncogene, is considered to be closely associated with the proliferation and metastasis of tumor cells. After polydatin treatment, the protein expression of c-Myc showed a significant decrease. Based on these data, we overexpressed c-Myc in cervical cancer cells and observed that the overexpression of c-Myc rescued the inhibitory effect of polydatin on cell proliferation and metastasis. These results indicated that polydatin can inhibit cell proliferation and metastasis through suppressing the c-Myc expression in human cervical cancer.

## Introduction

Among females, cervical cancer is the fourth most common malignancy in terms of incidence and mortality (Bray et al., 2018). The highest incidences occur in the developing parts of the world, where cervical cancer is the leading cause of cancer-related death among females in most countries (Jemal et al., 2011; Vaccarella et al., 2017). Cervical cancer is closely related to the high infection rate of human papillomavirus (HPV) infection (Hu and Ma, 2018). In addition, various biochemical alterations as well as genetic and epigenetic changes are associated with the initiation and development of cervical cancer (den Boon et al., 2015; Shafabakhsh et al., 2019). Although progress has been made in the prevention, diagnosis, and treatment of cervical cancer, the prognosis remains relatively poor (Marquina et al., 2018). Therefore, there is an urgent need for more effective treatment strategies for cervical cancer.

Recent studies have shown that stilbenes, such as resveratrol and pterostilbene, extracted from plants have a variety of antitumor activities. The effects and mechanisms of these stilbenes showed multiformity in different types of cancer. For instance, in non-small cell lung cancer (NSCLC), pterostilbene can decrease cell viability and induce apoptosis by activating endoplasmic reticulum stress signaling (Ma et al., 2017), while in ovarian cancer cells, pterostilbene can inhibit cell growth by inducing apoptosis and blocking cell cycle progression via inhibition of the STAT3 pathway (Wen et al., 2018). Increasing evidence has proven the antitumor effect of resveratrol. Notably, in human hepatocellular carcinoma cells, resveratrol suppressed invasion of cells through inhibition of tumor necrosis factor-α-mediated matrix metalloproteinase 9 (MMP9) expression (Yu et al., 2008) and caused cell cycle arrest via the activation of the p53-dependent pathway in colorectal cancer (Liu et al., 2019). These lines of evidence significantly suggested that stilbenes are promising candidates for tumor therapy.

As a stilbene, polydatin (PD) is an active ingredient from the roots of the traditional Chinese herb, *Polygonum cuspidatum*. There have been many studies on the pharmacological effects of PD in recent years. Previous studies on PD have mainly investigated its functions in cardiovascular (Xing et al., 2009; Zhang et al., 2015) and liver protection (Zhang et al., 2012; Xu et al., 2016), and it was not until recently that the antitumor effects of PD have received widespread attention. In NSCLCs, PD can suppress proliferation and metastasis via the NF-κB pathway (Zou et al., 2018). Additionally, in colorectal cancer, PD can effectively inhibit cell proliferation and promote cell apoptosis by regulating the miR-382/PD-L1 axis (Jin et al., 2020). However, the role of PD in cervical cancer remains to be further explored.

In this paper, we explored the role of PD in the proliferation and metastasis of cervical cancer. Our studies indicated that PD inhibited the proliferation, cell cycle progression, and migration/invasion of cervical cancer cells by mediating the c-Myc pathway. These findings suggested that PD may act as a potential candidate drug for the treatment of cervical cancer.

## Materials and Methods

### Cell Culture and Drug Treatment

Human cervical cancer cell lines (CaSki and C33A) and human embryonic renal cell line (293FT) were obtained from American Type Culture Collection (ATCC, Rockville, MD, United States). Both cervical cancer cell lines were cultured with 10% fetal bovine serum (FBS) and 1% penicillin-streptomycin (P/S) in DMEM (Thermo Fisher Scientific, Waltham, MA, United States). 293FT cells were cultured in DMEM additionally contained 1% G148 (Invitrogen, United States), 1% non-essential amino acids (Invitrogen, United States), 2% glutamine (Invitrogen, United States) and 1% sodium pyruvate (Invitrogen, United States). They were cultured with 5% CO_2_ at 37°C in a humidified incubator. Polydatin was obtained from MUST BIOTECHNOLOGY (Cheng Du, China) and was dissolved in dimethyl sulfoxide (DMSO; Sigma-Aldrich, Merck, Shanghai, China) as 100 mM stock solutions. Cervical cancer cells were treated with PD at the indicated time and concentrations.

### Lentivirus Production and Cell Infection

Human full-length c-Myc cDNA (NM_002467.6) was obtained from National Center for Biotechnology Information (NCBI), and the full-length c-Myc cDNA was amplified by PCR and ligated into PCDH-CMV-GFP-MCS-EF1-puro vector. Lentiviral production, infection, and establishment of stable human cervical cancer cell lines with the overexpression of c-Myc gene were performed as previously described (Zhao Y. et al., 2017).

### Cell Proliferation and Viability Assay

Cell proliferation was detected by MTT assay. Briefly, 2,000 cells/well were plated into 96-well plates and allowed to attach overnight at 37°C. After being incubated with dimethyl sulfoxide (DMSO) or PD at indicated concentrations for the indicated time, 20 μl of MTT [5 μg/ml of MTT in phosphate-buffered saline (PBS); Sigma] was added to each well and then incubated at 37°C for 2 h; the formazan complex was removed with DMSO, and the absorbance was measured with a wavelength of 560 nm using a microplate.

### BrdU Staining

In 6-cm dishes, 1 × 10^4^ cells were cultured and treated with DMSO or PD at indicated concentrations for 48 h, and then the BrdU assay was conducted as previously described (Zhao E. et al., 2017).

### Cell Cycle Assay

Cells were cultured in 6-cm dishes for 24 h and then treated with DMSO or PD at indicated concentrations. After 48 h of treatment, the cells were washed with cold PBS and then fixed in 70% ethanol at 4°C for more than 24 h. Subsequently, the cells were mixed with RNase A and stained with propidium iodide (PI; Beckman Coulter, United States) at room temperature for 30 min in the dark, and the cell cycle was tested with CytoFLEX (Beckman Coulter, United States).

### Wound-Healing Assay

Cells were cultured in 24-well plate and reached full confluence; then the monolayer of the cells was scratched with a white pipette tip. Subsequently, the floating and damaged cells were washed and removed with PBS, and serum-free medium containing DMSO or PD at indicated concentrations was added to culture the cells. The migrations of cells in the denuded area were observed, and pictures were taken at the indicated time.

### Transwell Migration and Invasion Assay

The migration assay was performed under the treatment of DMSO or PD at indicated concentrations as previously described (He et al., 2018).

### Western Blot Assay

After being treated with DMSO or PD at indicated concentrations, human cervical cancer cells were harvested, and the Western blot assay was performed as previously described (Zhang et al., 2018). All the primary antibodies used in this study are listed here: p21 (1:1,000, Cell Signaling Technology, United States), p27 (1:1,000, Cell Signaling Technology, United States), CDK2 (1:1,000, Cell Signaling Technology, United States), CDK4 (1:1,000, Cell Signaling Technology, United States), Cyclin D1 (1:1,000, Cell Signaling Technology, United States), Cyclin E1 (1:1,000, Cell Signaling Technology, United States), E-cadherin (1:1,000, Cell Signaling Technology, United States), N-cadherin (1:1,000, Cell Signaling Technology, United States), Snail (1:1,000, Cell Signaling Technology, United States), Slug (1:1,000, Proteintech, China), Vimentin (1:1,000, Proteintech, China), Tubulin (1:1,000, Cell Signaling Technology, China), and c-Myc (1:1,000, Abcam, United States). horseradish peroxidase (HRP)-labeled goat anti-mouse IgG (H + L) (A0216, 1:1,0000) or goat anti-rabbit IgG (H + L) (A0208, 1:1,0000) are used as a secondary antibody. Proteins were finally visualized by the electro-chemiluminescence (ECL) system (Beyotime, China) and then captured by Western blotting detection instruments (Clinx Science).

### Soft Agar Colony Formation Assay

Soft agar assays were conducted to further study the effect of PD on the cloning formation ability of cervical cancer cells, which was operated as previously described (Zhao E. et al., 2016).

### Tumor Xenografts

Twelve 4-week-old female nude mice (BALA/c) were purchased from Beijing Laboratory Animal Research Center and were domesticated in a specific pathogen-free (SPF) room. After 2 weeks, 1 × 10^6^ CaSki cells infected with c-Myc and empty vector in 100 μl of PBS were subcutaneously injected to flanks of mice (CaSki cells overexpressed with empty vector were on the left side; cells infected with c-Myc on the right). About 7 days after the injection, the mice were randomly divided into two groups. One group was then injected with PD (100 mg/kg), and the other was injected with DMSO as control every day for about 3 weeks. During this period, the tumor lengths and widths were measured, and the tumor volumes were calculated with the formula volume = (π/6) × length × width^2^. Finally, the mice were sacrificed, and formed tumors were removed and weighed. The execution of this study was under the approved guidelines. The protocol was pre-approved by the Animal Care and Use Committee of Southwest University. All works were made to minimize the suffering of the animals.

### Immunohistochemistry Staining

The immunohistochemistry staining assay was operated as previously reported (Yang R. et al., 2016). Rabbit Ki67 primary antibody (1:100, BD Biosciences, China) and rabbit c-Myc primary antibody (1:100, Abcam, United States) were used to achieve the corresponding antigen detection. The experiments were carried out independently three times.

### Statistical Analysis

All experiments were carried out with three technical and biological replicates. All the results acquired in this study are presented as the means ± standard deviation (SD). Differences between means were determined via unpaired Student’s *t-*tests, and *P* < 0.05 was considered statistically significant.

## Results

### Polydatin Inhibits Cervical Cancer Cell Growth and Proliferation

**FIGURE 1:**
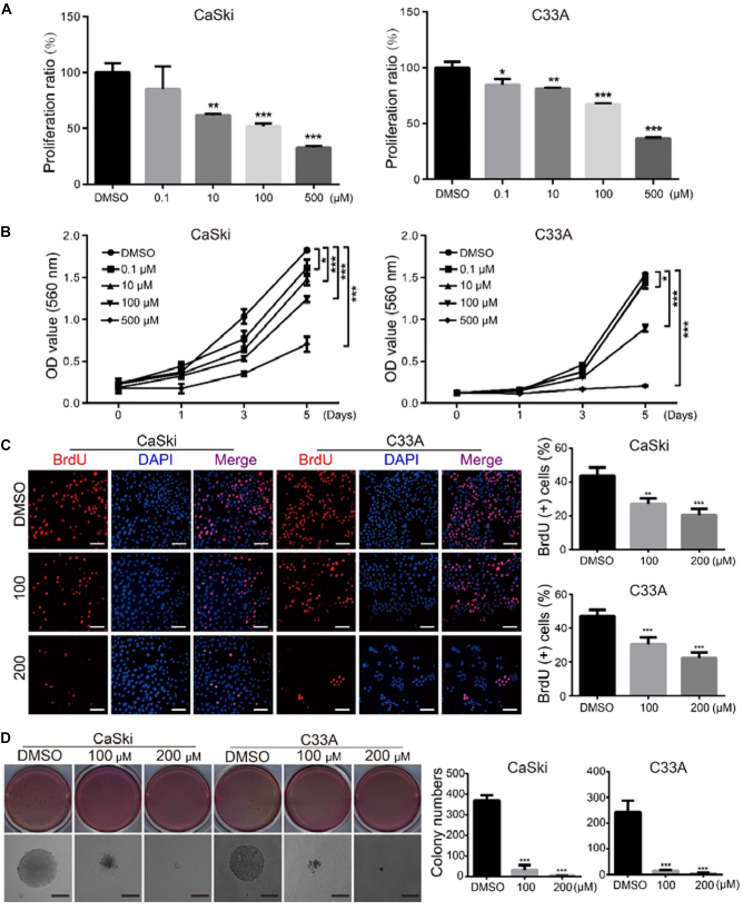
Polydatin inhibits cervical cancer cell growth and proliferation. **(A)** CaSki and C33A cervical cancer cells after treating with dimethyl sulfoxide (DMSO) or the indicated concentrations of polydatin for 72 h. The histogram demonstrates the quantification of proliferation ratio. DMSO was used as the control. **(B)** Cell growth was monitored using MTT assays in cells treated with polydatin at the indicated time and concentrations. **(C)** Immunofluorescence staining for BrdU was performed. DAPI was used for nuclear staining. Scale bar, 20 μm. The histogram demonstrates the quantification of the ratio of BrdU-positive cells. **(D)** The colony formation was examined by soft agar assays (1,000 cells/well) in CaSki and C33A cervical cancer cells after treating with DMSO or polydatin at the indicated concentrations for 14–21 days, Scale bar, 100 μm. All data were analyzed using two-tailed Student’s tests. Error bars, ^∗^*P* < 0.05, ^∗∗^*P* < 0.01, and ^∗∗∗^*P* < 0.001.

To evaluate the inhibitory effect of PD on the proliferation of human cervical cancer cells, CaSki and C33A cells were treated with different concentrations of PD for 72 h, and the results showed that the cell proliferation was inhibited in a dose-dependent manner (Figure 1A). To further confirm the inhibition, cell growth curve assays and BrdU staining were performed. The MTT data demonstrated that cell proliferation was significantly inhibited by PD in cervical cancer (Figure 1B). And the percent of BrdU-positive cells was lower in both cell lines after PD treatment for 48 h in a dose-dependent manner (Figure 1C). Moreover, soft agar assays were conducted to further study the effect of PD on the clone formation ability of cervical cancer cells. The results indicated that compared with the control group, the PD-treated group had smaller and fewer colonies (Figure 1D). These results described above indicated that PD markedly inhibited cervical cancer cell growth and proliferation.

### Polydatin Induces Cell Cycle Arrest at the G0/G1 Phase

**FIGURE 2:**
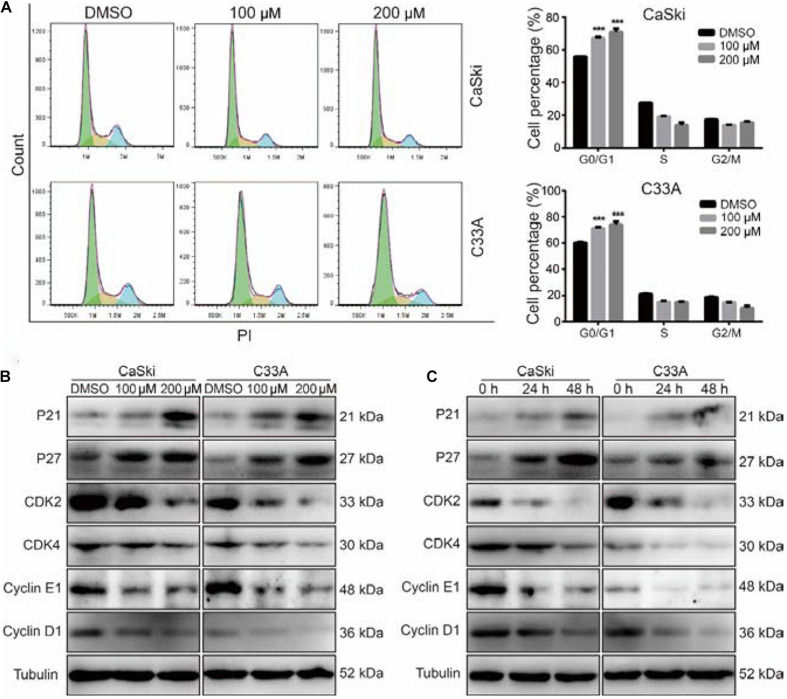
Polydatin inhibits cell growth by inducing cell cycle arrest at the G1/G0 phase. **(A)** Cell cycle analyses were performed using flow cytometry in CaSki and C33A cervical cancer cells after treating with dimethyl sulfoxide (DMSO) or polydatin at the indicated concentration for 48 h. The histogram demonstrates the quantification of the number of cells in different periods. **(B,C)** Western blot analysis of the cell cycle-related protein levels in CaSki and C33A cells. Cells were treated with polydatin at indicated concentrations or at indicated time. Tubulin was used as a control. All data were analyzed using two-tailed Student’s tests. Error bars, ^∗^*P* < 0.05, ^∗∗^*P* < 0.01, and ^∗∗∗^*P* < 0.001.

To evaluate whether PD inhibited cell growth and proliferation through cell cycle arrest, we used flow cytometry to detect the effect of PD on the cell cycle. The results suggested that the treatment of cervical cancer cells with PD induced significant G0/G1 phase arrest in a dose-dependent manner compared with DMSO treatment (Figure 2A). To explore the mechanism underlying this arrest, we measured the expression of p21, p27, CDK2, CDK4, Cyclin D1, and Cyclin E1 by Western blot. We noticed that the expression levels of p21 and p27 were upregulated and CDK4, CDK6, Cyclin D1, and Cyclin E1 were downregulated in the PD-treated cells in a dose- and time-dependent manner (Figures 2B,C). All these results indicated that PD caused cell cycle arrest at the G0/G1 phase in cervical cancer cells.

### Polydatin Inhibits Cervical Cancer Cell Migration and Invasion

**FIGURE 3:**
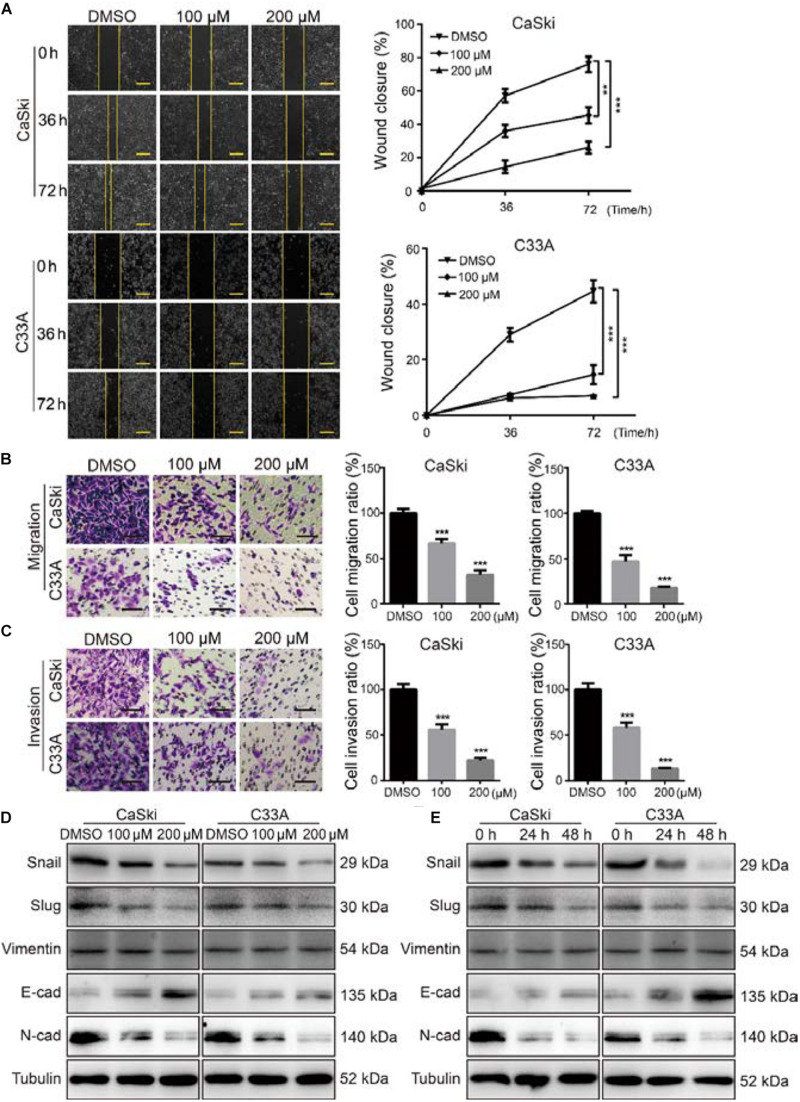
Polydatin inhibited cervical cancer cell migration and invasion. **(A)** The migration by wound-healing assays of CaSki and C33A cells after treating with dimethyl sulfoxide (DMSO) or polydatin at the indicated concentrations for the indicated time. Scale bar, 100 μm. **(B)** The effect of Transwell migration assays in CaSki and C33A cells after treating with DMSO or polydatin at the indicated concentrations for 24 h. Scale bar, 100 μm. **(C)** The effect of Transwell invasion assays in CaSki and C33A cells after treating with DMSO or polydatin at the indicated concentrations for 48 h. Scale bar, 100 μm. **(D,E)** Western blot analysis of the EMT-related protein levels in CaSki and C33A cells. Cells were treated with polydatin at indicated concentrations or at indicated time. Tubulin was used as a control. All data were analyzed using two-tailed Student’s tests. Error bars, ^∗^*P* < 0.05, ^∗∗^*P* < 0.01, and ^∗∗∗^*P* < 0.001.

As the metastasis of cervical cancer is closely correlated with poor prognosis and poor therapeutic outcomes (Wang and Chen, 2019), we further investigated the effect of PD on the migration and invasion abilities of cervical cancer cells. The wound healing assays indicated that cervical cancer cells treated with PD exhibited markedly reduced wound closure ratios than did control cells (Figure 3A). Similarly, Transwell migration assays further indicated that cervical cancer cells treated with PD showed significantly inhibited cell migration ability compared with the DMSO-treated cells (Figure 3B). Furthermore, we utilized Transwell invasion assays to evaluate the effect of PD on cell invasion. The results showed that PD treatment prominently decreased the number of cells that penetrated the Matrigel-coated membrane (Figure 3C). To confirm these results, we measured the expression of E-cadherin, N-cadherin, Snail, Slug, and Vimentin by Western blot. The expression of E-cadherin, an epithelial marker, was upregulated, and the expression of Vimentin was not changed after PD treatment (Figures 3D,E). But the expressions of N-cadherin, Snail, and Slug were downregulated in a dose- and time-dependent manner (Figures 3D,E). Taken together, these findings indicated that PD treatment reversed the epithelial–mesenchymal transition (EMT) of cervical cancer cells. Therefore, these findings significantly indicated that PD could suppress cervical cancer cell migration and invasion.

### Polydatin Inhibits Cell Growth and Proliferation by Suppressing the c-Myc Expression

**FIGURE 4:**
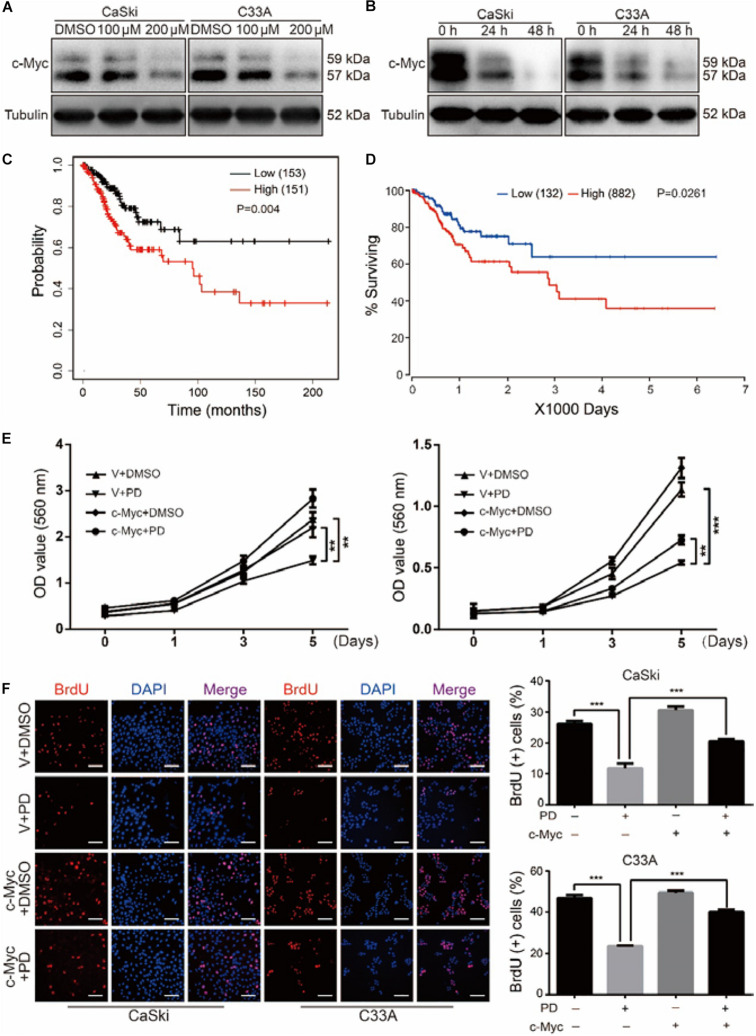
Polydatin inhibits cell growth and proliferation through suppressing the c-Myc expression. **(A,B)** Western blot assays were used to show the expression of c-Myc after dimethyl sulfoxide (DMSO) or polydatin treatment. Cells were treated with polydatin at indicated concentrations or at indicated time. Tubulin was used as a control. **(C,D)** Kaplan–Meier overall survival for c-Myc expression in cervical cancer tumors (KM plotter dataset and OncoLnc dataset). **(E)** The effect of DMSO or 200 μM of polydatin on the viability of c-Myc/vector-overexpressed CaSki and C33A cells using MTT assays. **(F)** Image and quantification of c-Myc-overexpressed CaSki or C33A cells as well as vector cells positive for BrdU staining after treating with DMSO or 200 μM of polydatin for 48 h. Scale bar, 100 μm. All data were analyzed using two-tailed Student’s tests. Error bars, ^∗^*P* < 0.05, ^∗∗^*P* < 0.01, and ^∗∗∗^*P* < 0.001.

After treating with indicated concentrations of PD for 48 h, we found that the important transcription factor protein c-Myc in both CaSki and C33A cells was notably decreased in a dose-dependent manner. Moreover, CaSki and C33A cells were treated with 200 μM of PD for 24 and 48 h. Similarly, the results showed that c-Myc was reduced in a time-dependent manner (Figures 4A,B). These findings suggested that PD might inhibit cell proliferation and metastasis through suppressing the c-Myc expression. To investigate whether c-Myc has a prognostic value for cervical cancer, we assessed the prognostic value of c-Myc expression through two publicly available clinical tools, as follows: Kaplan–Meier Plotter^1^ and OncoLnc.^2^ Progression-free survival Kaplan–Meier analysis of these two databases suggested that high expression of c-Myc was significantly correlated with poor overall survival probability, whereas low expression was significantly correlated with good prognosis (Figures 4C,D).

CaSki and C33A cells were then stably infected with lentivirus encoding c-Myc. We further studied the cell growth curve by MTT assays for 5 days in c-Myc/empty vector-overexpressed CaSki and C33A cells after the treatment of PD at indicated concentration or DMSO. The results revealed that the overexpression of c-Myc promoted cell proliferation and notably decreased cell proliferation inhibition caused by PD (Figure 4E). The BrdU assays also showed that the decreased proliferation ability caused by PD was rescued in c-Myc-overexpressed cells compared with empty vector-overexpressed cells (Figure 4F). Taken together, these findings suggested that the overexpression of c-Myc could reverse the inhibition of proliferation caused by PD in human cervical cancer cells.

### The Upregulation of c-Myc Reverses Polydatin-Induced Cell Cycle Arrest

**FIGURE 5:**
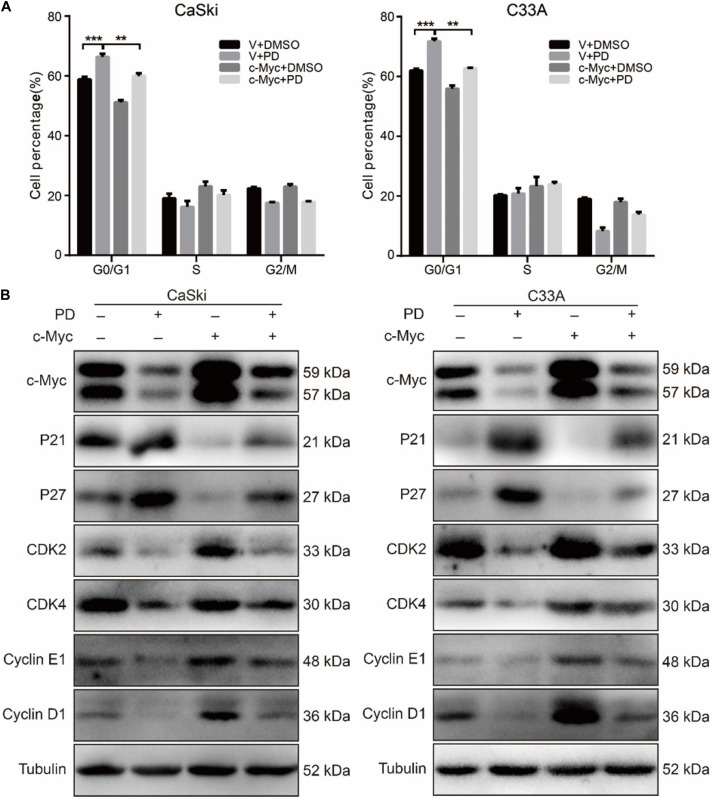
The upregulation of c-Myc rescues polydatin-induced cell cycle arrest. **(A)** The cell cycle of CaSki and C33A cells was analyzed by flow cytometry in c-Myc-overexpressed cells as well as vector cells after being treated with dimethyl sulfoxide (DMSO) or 200 μM of polydatin for 48 h. **(B)** Western blot analysis of cell cycle-related proteins in c-Myc-overexpressed CaSki and C33A cells as well as vector cells after being treated with DMSO or 200 μM of polydatin for 48 h. Tubulin was used as a control. All data were analyzed using two-tailed Student’s tests. Error bars, ^∗^*P* < 0.05, ^∗∗^*P* < 0.01, and ^∗∗∗^*P* < 0.001.

To further study the PD-induced cell cycle arrest, we treated c-Myc- and empty vector-overexpressed cervical cancer cells with 200 μM of PD for 48 h, and we examined the cell cycle using flow cytometry. The results indicated that c-Myc-overexpressed CaSki and C33A cells showed a decline in the percentage of G0/G1 phase compared with the empty vector-overexpressed group after PD treatment (Figure 5A), which meant overexpression of c-Myc reversed the cell cycle arrest induced by PD. Then, we measured the expression of cell cycle-related proteins for the G0/G1 phase, including p21, p27, CDK2, CDK4, Cyclin D1, and Cyclin E1. We observed that compared with the empty vector expressing cells, CaSki and C33A cells overexpressing c-Myc and treated with 200 μM of PD for 48 h showed downregulation of p21 and p27 and upregulation of CDK2, CDK4, Cyclin D1, and Cyclin E1 (Figure 5B). These results suggested that the upregulation of c-Myc could reverse the cell cycle arrest induced by PD.

### The Upregulation of c-Myc Restores the Cell Migration and Invasion Inhibited by Polydatin

**FIGURE 6:**
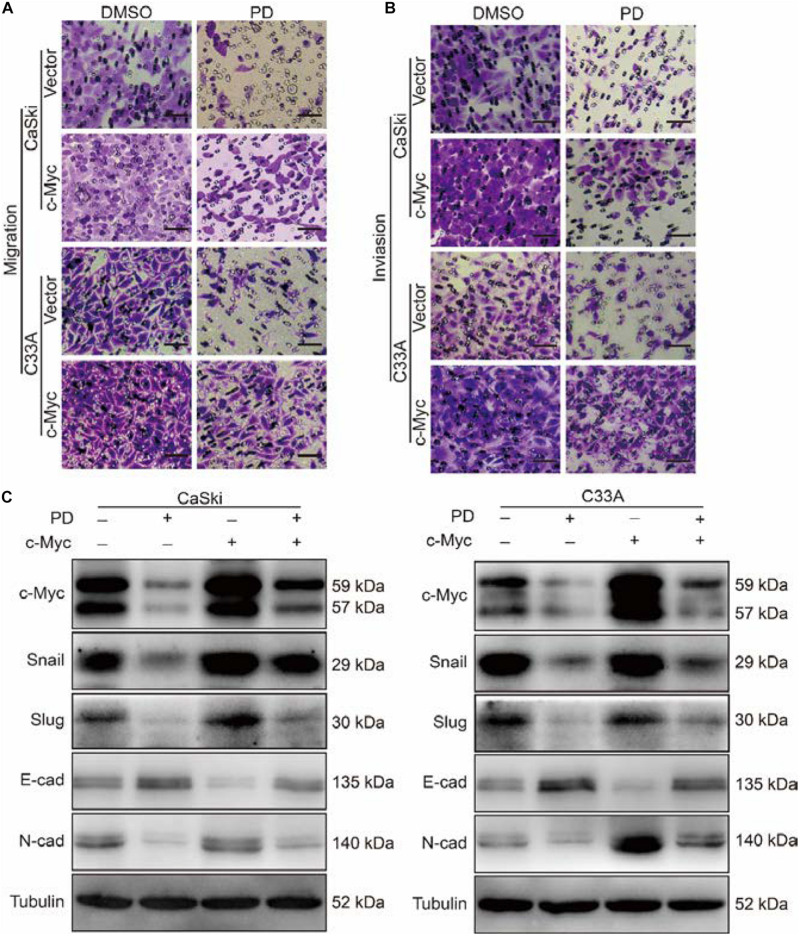
The upregulation of c-Myc retrieved cell migration and invasion inhibited by polydatin. **(A,B)** The effect of the Transwell migration and invasion assays in c-Myc-overexpressed CaSki or C33A cells as well as vector cells after treating with dimethyl sulfoxide (DMSO) or 200 μM of polydatin for 24 and 48 h. Scale bar, 100 μm. **(C)** Western blot assays were used to show the expression of epithelial–mesenchymal transition (EMT)-related proteins in c-Myc-overexpressed CaSki and C33A cells as well as vector cells after being treated with DMSO or 200 μM of polydatin for 48 h. Tubulin was used as a control. All data were analyzed using two-tailed Student’s tests. Error bars, ^∗^*P* < 0.05, ^∗∗^*P* < 0.01, and ^∗∗∗^*P* < 0.001.

As c-Myc was reported to be associated with tumor metastasis (Zhang et al., 2005; Planas-Silva et al., 2007), we further studied the effect of PD on the migration and invasion abilities of cervical cancer cells after c-Myc overexpression. Through Transwell migration and invasion experiments, we found that the migration ability was partly restored in PD-treated cells after c-Myc overexpressed (Figure 6A and Supplementary Figure 1A) and retrieved the effects of PD-induced invasive inhibition compared with the empty vector-overexpressed group with PD treatment (Figure 6B and Supplementary Figure 1B). To confirm these results, we evaluated EMT-related proteins, which are important markers of cell metastasis. The upregulation of c-Myc significantly reduced the PD-induced upregulation of E-cadherin and downregulation of N-cadherin, Snail, and Slug (Figure 6C), which indicated the important role of c-Myc in PD-induced EMT inhibition. These results suggested that the upregulation of c-Myc could restore the cell migration and invasion inhibited by PD.

### The Upregulation of c-Myc Relieves the Polydatin-Induced Inhibition of Clonogenicity and Tumorigenesis

**FIGURE 7:**
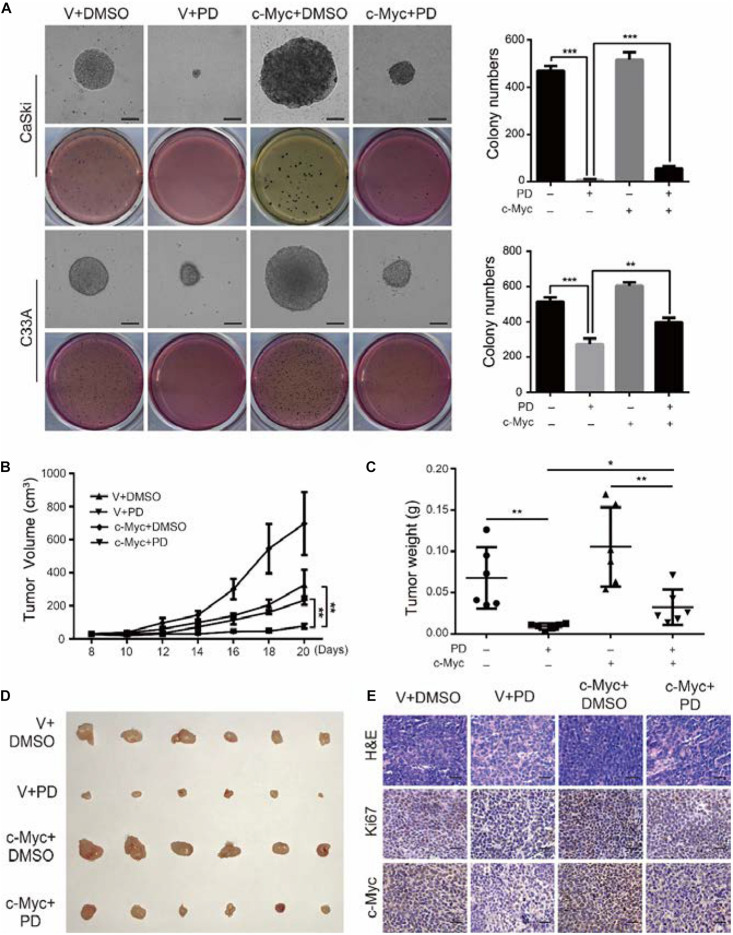
The upregulation of c-Myc relieves the polydatin-induced inhibition of clonogenicity and tumorigenesis. **(A)** The colony-formation ability of c-Myc-overexpressed cells was detected after treating with DMSO or 200 μM of polydatin. Scale bar, 100 μm. Colony numbers in the panel were quantified. **(B,C)** Tumor volumes and weights of indicated tumors were recorded and analyzed. **(D)** Photograph of tumors from indicated mice. **(E)** H&E staining and immunohistochemical (IHC) staining of Ki67 and c-Myc at indicated tumors. Scale bar, 100 μm. Dimethyl sulfoxide (DMSO) and empty vector (V) were used as control. All data were analyzed using two-tailed Student’s tests. Error bars, ^∗^*P* < 0.05, ^∗∗^*P* < 0.01, and ^∗∗∗^*P* < 0.001.

We have demonstrated that PD treatment could inhibit the clonogenicity of cervical cancer cells. To further analyze the effect of c-Myc on clonogenicity, we conducted soft agar assays. The results showed that after PD treatment, compared with the empty vector expression group, the c-Myc overexpression group contained larger and more numerous colonies (Figure 7A). These results indicated that the inhibition of PD can be reversed by the overexpression of c-Myc in human cervical cancer. Besides, CaSki cells were transplanted subcutaneously into 4-week-old female nude mice. The results showed that the tumor volumes and weights of the PD treatment group were significantly smaller than those of the DMSO treatment group, and tumor growth was significantly inhibited by PD. In addition, after PD injection, mice transplanted with c-Myc-overexpressed CaSki cells showed larger tumor volumes and weights and a faster growth rate than the mice injected with empty vector-overexpressed CaSki cells (Figures 7B–D), which suggested that PD can inhibit tumorigenesis *in vivo* and that the inhibition can be reversed by overexpression of c-Myc. Hematoxylin and eosin (H&E) staining and immunohistochemical (IHC) staining with Ki67 and c-Myc were conducted and supported the results described above (Figure 7E). In summary, the upregulation of c-Myc could relieve the PD-induced inhibition of clonogenicity and tumorigenesis.

## Discussion

Cervical cancer is one of the most common gynecological malignancies, which ranks fourth among malignant tumours in females in terms of both new cases and the proportion of cancer-related deaths (Bray et al., 2018). Because of the high cost, high invasiveness, and low efficacy of cervical cancer treatments, it is necessary to find new potential drugs and therapies to further improve the treatment of cervical cancer. Stilbenes are a novel treatment strategy that has become an effective and promising treatment option for many types of cancer (Yu et al., 2008; Ma et al., 2017; Wen et al., 2018; Liu et al., 2019). The stilbene PD has been shown to inhibit cell proliferation in colorectal cancer and suppress the metastatic potential of NSCLC cells (Zou et al., 2018; Jin et al., 2020). However, the effect of PD in cervical cancer is less well studied.

In this study, we tried to evaluate the effects of PD in cervical cancer. The MTT assays indicated that PD significantly inhibited cell proliferation in a dose- and time-dependent manner. The BrdU assays showed that the numbers of BrdU-positive cells were significantly reduced. Soft agar experiments showed the formed colonies after PD treatment became increasingly smaller. Xenograft experiments indicated that the tumors formed in nude mice exhibited slower growth and smaller size after PD treatment. The weights of tumors were also significantly reduced. These findings indicated that PD could inhibit the proliferation of cervical cancer cells both *in vitro* and *in vivo*.

Then, through wound healing and Transwell assays, we found that PD could markedly inhibit cervical cancer cell migration and invasion. EMT is a process in which epithelial cells lose cell polarity and intercellular contacts, showing remarkable morphological alterations (Nishioka et al., 2010). In this process, epithelial cells lose their cell adhesion and epithelial components and acquire migratory and mesenchymal phenotypes (Cercelaru et al., 2017). We assessed the expression of EMT-related proteins, such as E-cadherin, N-cadherin, Snail, Slug, and Vimentin, after PD treatment. The experimental results showed increased expression of E-cadherin and decreased expression of N-cadherin, Snail and Slug. These findings suggested that PD inhibited EMT in cervical cancer cells. All the evidences described above indicated that PD could inhibit migration and invasion of cervical cancer cells by reversing EMT progression.

The proto-oncogene c-Myc is the homolog of the v-Myc in human cells and has been discovered to be a strong transcription factor (Huang et al., 2016). In the process of normal cell proliferation, the expression of c-Myc is strictly controlled, but in malignancies of different histogenetic origins, the increased c-Myc expression is often observed. In cervical cancer, recent reports have indicated that the c-Myc expression increases with increasing severity of histological diagnosis (Heng et al., 2018; Ji et al., 2018); these results emphasize the potential function of c-Myc as an indicator in cervical cancer (Li et al., 2014; Gao et al., 2015; Zhao W. H. et al., 2016). In this study, the expression of the proto-oncogene c-Myc was notably decreased in a dose- and time-dependent manner at both the mRNA and protein levels in two human cervical cancer cell lines (CaSki and C33A) after PD treatment. Furthermore, c-Myc can affect the expression of various proteins that are associated with the cell cycle and metastasis through transcriptional regulation and post-transcriptional modification pathways (Nowak et al., 2015; Dejure and Eilers, 2017; Chen et al., 2018; Baluapuri et al., 2020; Thevenon et al., 2020). Based on these results, we speculated that PD inhibits the expression levels of cell cycle-related proteins and EMT-related proteins by downregulating c-Myc, thereby inhibiting the proliferation and metastasis abilities of cervical cancer cells. To confirm this hypothesis, we overexpressed c-Myc in CaSki and C33A cells. MTT assays and related experiments indicated that overexpressing c-Myc partially reversed the PD-mediated inhibition of cell growth and proliferation based on the recovery of cell cycle and cell cycle-related protein expression. In the process of cell proliferation, c-Myc can control the progress of the cell cycle by regulating various cell cycle-related proteins (García-Gutiérrez et al., 2019). Among these mechanisms, one of the main ways that c-Myc regulates the cell cycle is through inhibition of p21 transcription and induction of p27 degradation (Bretones et al., 2015). The p21 is a common tumor suppressor gene that can cause cell cycle arrest in the G1 phase (Liu et al., 2010; Karimian et al., 2016; García-Gutiérrez et al., 2019). The proto-oncogene c-Myc can inhibit the regulatory effect of p21 on the cell cycle by inhibiting its promoter (Bretones et al., 2015). And p27 is also a tumor suppressor gene that can inhibit the progression of cells from the G1 to S phase (Abbastabar et al., 2018; García-Gutiérrez et al., 2019). Previous studies have shown that c-Myc can regulate p27 by inhibiting its transcriptional promoter and promote p27 degradation by post-transcriptional regulation (Bretones et al., 2015).

**FIGURE 8:**
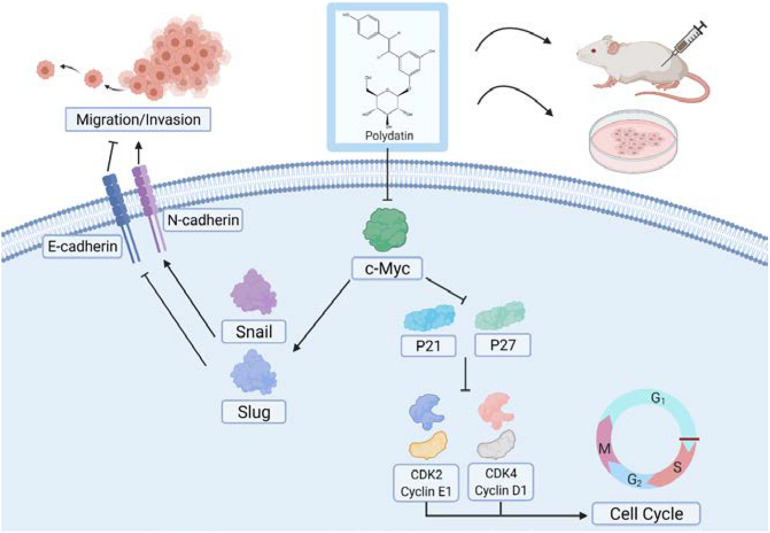
Model for polydatin inhibition of cell proliferation and metastasis of human cervical cancer.

Cyclin dependent kinases (CDKs) are a class of serine/threonine protein kinases that cooperate with cyclin and are important factors in cell cycle regulation (Finn et al., 2016). A specific CDK can combine with a specific cyclin to form a heterodimer, and different cyclin–CDK complexes catalyze the phosphorylation of different substrates through a specific CDK activity, thereby promoting and transforming the cell cycle (Finn et al., 2016). During the cell cycle, Cyclin D1 promotes cell proliferation by binding to CDK4 to form a complex. However, p21 can bind to CDK4 to block its interaction with Cyclin D1, resulting in G1/S phase cell cycle arrest (Thomasova and Anders, 2014). Additionally, p21 can also inhibit other cell cycle complexes, such as Cyclin E1/CDK2 to regulate the cell cycle (Karimian et al., 2016). Cyclin E usually binds to CDK2 to form a complex to promote cell cycle progression. CDK2 is the main target of p27. p27 can inhibit CDK2 through its kinase binding region, thereby blocking the interaction of the Cyclin E1/CDK2 complex and affecting the progression of the cell cycle (Aleem et al., 2005; Abbastabar et al., 2018). In this study, we believe that PD may promote p21 and p27 in cells by inhibiting the c-Myc signaling pathway, thereby inhibiting the expression of CDK and cyclin proteins and ultimately inhibiting cell proliferation activity (Figure 8). In addition, related studies have shown that the c-Myc protein itself can promote the expression of a variety of CDK and cyclin proteins at the transcriptional level (Bretones et al., 2015; Stine et al., 2015). Therefore, the inhibition of CDKs and cyclins proteins may also be a direct result of c-Myc inhibition mediated by PD.

In addition, cell migration and invasion assays showed that overexpressing c-Myc can partially reverse PD-mediated inhibition of cell migration and invasion and EMT-related proteins. The transcription factor Snail and Slug plays an integral role throughout EMT, which ultimately represses E-cadherin expression and increases the expression of N-cadherin (Batlle et al., 2000; Cano et al., 2000; Hao et al., 2012; Singh et al., 2018). Relevant studies have shown that c-Myc can enhance the gene transcription level of Snail and Slug (Cho et al., 2015; Yang X. et al., 2016; Wang et al., 2018; Cui et al., 2019), which may explain the phenomenon that Snail and Slug levels changed with c-Myc in this study. And the changes in the expression of E-cadherin and N-cadherin may be caused by the Snail and Slug. In this study, we hypothesized that PD may suppress Snail and Slug expression in cells by inhibiting the c-Myc signaling pathway, thereby increasing the expression of E-cadherin and inhibiting the expression of N-cadherin and ultimately inhibiting the cell metastasis capability (Figure 8).

In conclusion, the results presented here demonstrate that PD inhibits proliferation and metastasis through suppressing the c-Myc expression in cervical cancer and might be potential neoadjuvant chemotherapy or an alternative strategy for treating cervical cancer patients.

## Data Availability Statement

The original contributions presented in the study are included in the article/Supplementary Material, further inquiries can be directed to the corresponding author/s.

## Ethics Statement

The animal study was reviewed and approved by Institutional Animal Care and Use Committee of Southwest University.

## Author ContRibutions

EZ and HC designed the experiments, revised the manuscript, and approved the submission. LB, YM, XW, QF, and ZZ performed the experiments, analyzed the data, and wrote the manuscript. SW, HZ, XL, and YX prepared the samples and performed the experiments. All authors have read and agreed to the published version of the manuscript.

## Conflict of Interest

The authors declare that the research was conducted in the absence of any commercial or financial relationships that could be construed as a potential conflict of interest.

## Footnotes

1 http://kmplot.com

2 http://www.oncolnc.org/

## Abbreviations

ATCC: American Type Culture Collection
BrdU: 5-bromo-2-deoxyuridine
CDK: cyclin dependent kinase
CDK2: cyclin dependent kinase 2
CDK4: cyclin dependent kinase 4
DMSO: dimethyl sulfoxide
ECL: electro-chemiluminescence
EMT: epithelial–mesenchymal transition
FBS: fetal bovine serum
H&E: hematoxylin and eosin
HPV: human papillomavirus
HRP: horseradish peroxidase
IgG: immunoglobulin G
IHC: immunohistochemistry
V: empty vector
MMP9: matrix metalloproteinase-9
MTT: 3-(4,5-dimethylthiazol-2-yl)-2,5-diphenyl tetrazolium bromide
NCBI: National Center for Biotechnology Information
NF-κB: nuclear factor kappa-B
NSCLC: non-small cell lung cancers
P/S: penicillin-streptomycin
PBS: phosphate-buffered saline
PCR: polymerase chain reaction
PD: polydatin
PI: propidium iodide
PVDF: polyvinylidene difluoride
E-cad: E-cadherin
N-cad: N-cadherin.
