# Supplementary material for: Polydatin Inhibits Cell Viability, Migration, and Invasion Through Suppressing the c-Myc Expression in Human Cervical Cancer
Source: Front Cell Dev Biol. 2021 Apr 12;9:587218. doi: 10.3389/fcell.2021.587218 (PMC8072354; doi:10.3389/fcell.2021.587218)
Supplement: Supplementary file 1 [file Image_1.pdf]

Supplementary Figure

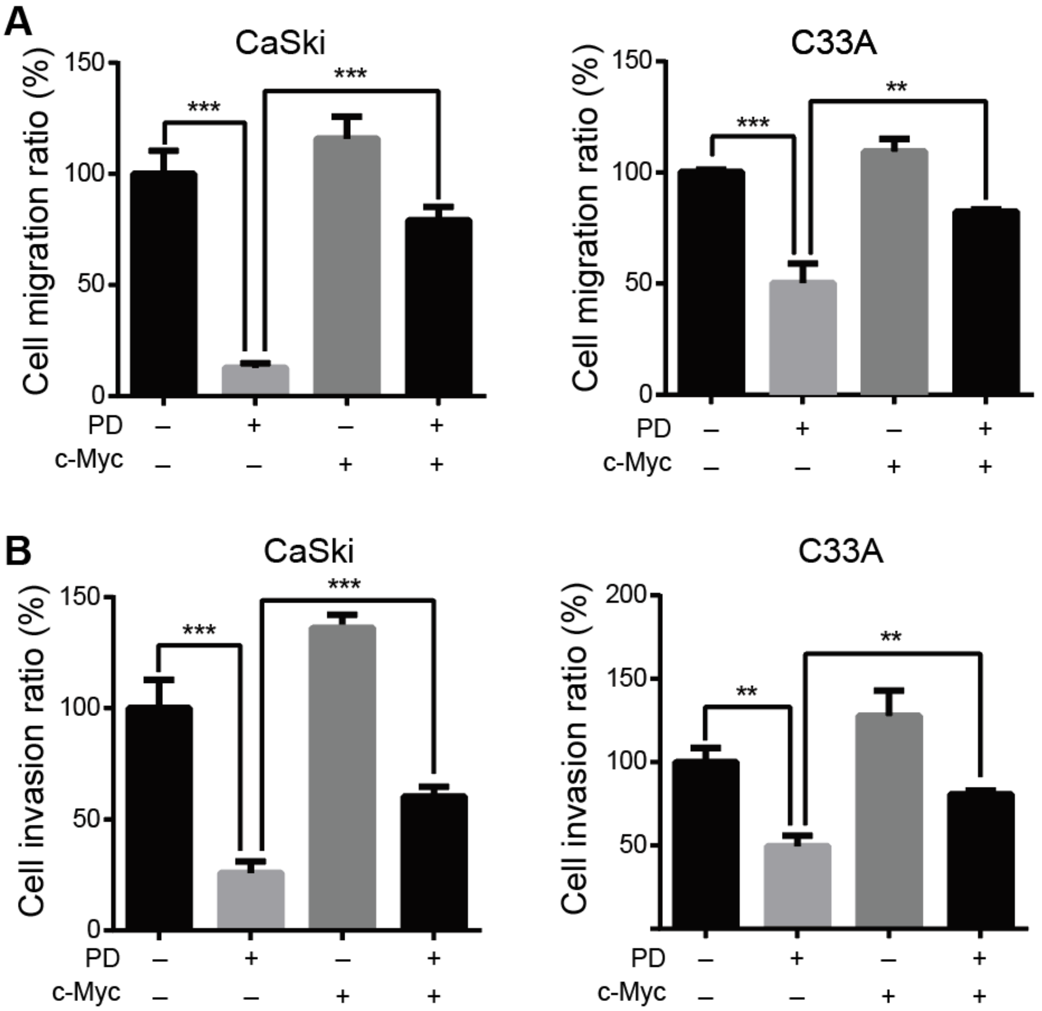

Supplementary Figure 1 The effect of the Transwell migration and invasion assays in c-Myc-overexpressed cancer cells treated with PD. **(A)** Migration ratios were normalized in c-Myc-overexpressed CaSki or C33A cells as well as vector cells after treating with DMSO or 200 μM polydatin for 24 h and 48 h respectively. **(B)** Invasion ratios were normalized in c-Myc-overexpressed CaSki or C33A cells as well as vector cells after treating with DMSO or 200 μM polydatin for 24 h and 48 h respectively
